# Biomarkers of oxidative status: missing tools in conservation physiology

**DOI:** 10.1093/conphys/cou014

**Published:** 2014-05-10

**Authors:** Michaël Beaulieu, David Costantini

**Affiliations:** 1 Zoological Institute and Museum, University of Greifswald, Johann-Sebastian Bach Straße 11/12, 17489 Greifswald, Germany; 2 Department of Biology, University of Antwerp, Campus Drie Eiken, DC 135, Universiteitsplein 1, 2610 Wilrijk, Antwerp, Belgium; 3 Institute for Biodiversity, Animal Health and Comparative Medicine, University of Glasgow, Glasgow G12 8QQ, UK

**Keywords:** Biomarker, conservation, ecophysiology, environmental disruption, oxidative stress, stress

## Abstract

Recent ecological studies have shown that oxidative status could have a significant impact on fitness components in wild animals. Not only can oxidative status reflect the environmental conditions that animals experience, but it can also predict their chances of reproduction and survival in the future in their natural habitat. Such important characteristics make markers of oxidative status informative tools to evaluate *a priori* individual perspectives of reproduction and survival as well as to assess *a posteriori* the effect of human activities on the fitness of species of conservation concern and wildlife in general. Markers of oxidative status may therefore help conservation practitioners to identify conservation threats to animal populations and to maximize the success of wildlife management. Despite these potential benefits for animal conservation programmes, up to now markers of oxidative status have only been reported anecdotally in conservation studies. The aim of this review is therefore to raise awareness by conservation practitioners of the use of markers of oxidative status. Towards this end, we first describe how environmental disruptions due to human activities can translate into variation in oxidative status. Second, we show how individual and population variation in oxidative status may contribute to the success or the failure of reintroduction or translocation programmes. Finally, we emphasize the technical features specific to the measurement of markers of oxidative status in conservation programmes, which may help investigators with the interpretation of their results. Such prior knowledge about markers of oxidative status may encourage conservation physiologists to use them in order to enhance the success of conservation programmes and wildlife management.

## Abstract (precis)

The use of oxidative status markers in animal conservation is dual-faceted, as they reflect the effects of environmentally-induced stress on animal populations and they predict individuals' fitness prospects. Paradoxically, conservation studies rarely include them. Here, we raise awareness of their use, and show how they can valuably complement conservation studies.

## Introduction

In the last 50 years, environmental conditions have changed at an unprecedented rate, impacting heavily on ecological processes (Diffenbaugh and Field, 2013). It is increasingly recognized that a large quota of these changes is due to human activities, which can have immediate effects on habitats, such as urbanization and deforestation, or more permanent and pernicious effects, such as habitat contamination and climate change. When facing such environmental disruptions, only three options are possible for animals: (i) adapt locally; (ii) leave in order to find more suitable conditions; or (iii) become extinct. Irrespective of the response adopted, environmental disruptions are paralleled by physiological changes that may be indicative of the capacity of animals to cope with the new conditions they encounter.

**Figure 1:**
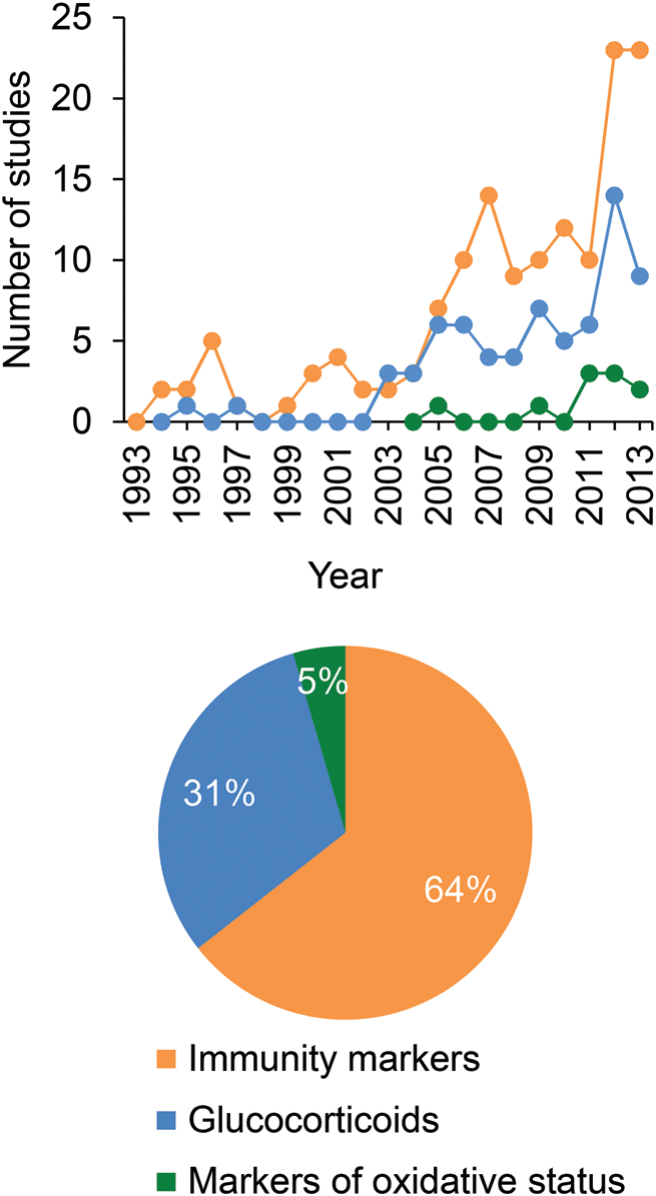
Number of studies including immunity parameters (orange), glucocorticoids (blue) or markers of oxidative stress (green) in view of conservation issues. The research was done in January 2014 by using Web of Knowledge and entering the following keywords: (i) “animal conservation” AND “animal population*” AND “immunity* OR immune*”; (ii) “animal conservation” AND “animal population*” AND “glucocorticoid* OR cortisol* OR corticosterone”; and (iii) “animal conservation” AND “animal population*” AND “oxidative stress* OR antioxidants*”. The upper panel represents the number of publications in each year, while the lower panel represents the relative contribution of each type of study.

Presumably because the hypothalamic–pituitary–adrenal axis first integrates environmental disruptions in vertebrates, stress hormones (glucocorticoids) have become key biomarkers to assess the physiological response of animals to these environmental disruptions (Romero, 2004; Wingfield, 2013) and have regularly been used as biomarkers of animal population health in conservation studies (Busch and Hayward, 2009; Fig. 1). The main action of stress-induced glucocorticoids is to redirect energy allocation towards immediate survival, through their stimulatory effects on metabolism. For instance, an increase in glucocorticoid levels results in the mobilization of stored energy, which can subsequently be used to increase foraging effort and food access (Romero, 2004). However, such energy diversion can occur only at the expense of other physiological functions; for instance, through decreased reproductive capacity or immunosuppressive effects (Romero, 2004; Wingfield, 2013).

Due to the stimulatory effects of glucocorticoids on metabolism, another potential cost of the stress response is an increased production of reactive oxygen species (ROS). Reactive oxygen species are byproducts of the aerobic metabolism of mitochondria or of other functions within the organism, such as immune cell activity. If left uncontrolled by antioxidant mechanisms, ROS can generate oxidative damage to biological compounds. Such an imbalance between ROS production and antioxidant defences resulting in oxidative damage has been termed ‘oxidative stress’ (Sies, 1991; Halliwell and Gutteridge, 2007). Different biomarkers can be used to quantify oxidative damage (Table 1; see also section on ‘*Methods and interpretation*’). Measuring levels of oxidative stress is highly fitness relevant, because high oxidative stress levels may compromise survival and reproduction (Costantini, 2008; Monaghan *et al.*, 2009). For instance, common yellowthroats (*Geothlypis trichas*) that do not survive from one year to another exhibit higher levels of oxidative damage than individuals that are able to survive (Freeman-Gallant *et al.*, 2011). Likewise, male great tits with higher levels of oxidative damage in their sperm have lower fertility (Helfenstein *et al.*, 2010). Owing to its significant effects on fitness components, oxidative stress is likely to impinge on the health of animal populations (Beaulieu *et al.*, 2013). To avoid these fitness-adverse effects, animals have the possibility of producing and/or mobilizing antioxidant defences of endogenous and/or exogenous origin (Table 2). Importantly, oxidative stress does not only occur following a stress response through elevated glucocorticoid levels, but it may also be triggered by other physiological (e.g. immune response), physical (e.g. heat) and/or chemical factors (e.g. pesticides) that can directly elevate ROS production (see below in the section ‘*A posteriori* effects of environmental disruptions on markers of oxidative status’ for a list of anthropogenic factors that may generate oxidative stress). Classical research on glucocorticoids has also made a clear distinction between acute and chronic exposure, because of the different consequences for individual behaviour and physiology (Romero, 2004). Such a distinction between acute and chronic is also valid for markers of oxidative status. For example, a recent meta-analysis showed that oxidative stress increased with an increase in the duration of physiological stress, while acute exposure mostly resulted in up-regulation of the antioxidant response (Costantini *et al.*, 2011a).

**Table 1:** Summary of methods that can be used to determine levels of oxidative damage in conservation studies, from biological matrices that do not require terminal sampling

Method	Biological matrix	Description
Hydroperoxides	Serum, plasma, tissue biopsy	Hydroperoxides derive from the early oxidation of several biomolecular substrates, such as polyunsaturated fatty acids, cholesterol, proteins and nucleic acids, and can be precursors of end-products of lipid peroxidation, such as MDA, HNE and isoprostanes. Colorimetric assays can be used to measure their concentration in a biological matrix
End-products of lipid damage (MDA, HNE, isoprostanes)	Serum, plasma, immune cells, urine, seminal plasma, tissue biopsy	These include several kinds of molecules, such as MDA, HNE and isoprostanes. High-performance liquid chromatography or GC-MS analysis is commonly used for their determination. Enzyme-linked immunosorbent assays are also available for isoprostanes; however, determination of isoprostanes is expensive and requires specialized personnel. Enzyme-linked immunosorbent assays are available for the determination of adducts between proteins and MDA or HNE (see protein carbonyls)
Oxidative protein damage	Serum, plasma, red blood cells, seminal plasma, tissue biopsy	Protein carbonyls derive from damage to proteins. Carbonyls (C = O) are introduced into proteins from free radicals or via reactions with lipid peroxidation products. Enzyme-linked immunosorbent assays, HPLC or electrophoresis/western blot are commonly used methods for the quantification of total protein carbonyls or of certain protein carbonyls, such as those derived from reaction with end-products of lipid peroxidation (MDA and HNE)
Thiobarbituric acid reactive substances	Serum, plasma, red blood cells, immune cells, urine, seminal plasma, tissue biopsy, yolk	It is not specific of a certain kind of damage; however, it provides a general quantification of oxidative damage molecules that is very sensitive to exposure of the organism to environmental stressors (e.g. contamination, those that elicite an increase in stress hormones)
Oxidative DNA damage molecules	Serum, plasma, red blood cells, urine, tissue biopsy	Various methods are available for the determination of a number of DNA damage compounds. These include HPLC or GC-MS, which requires specialized personnel; ELISAs are also available
DNA strand breakage	Lymphocytes, tissue biopsy	The comet assay is the classical method for the determination of DNA strand breakage. Under an electrophoretic field, damaged DNA is separated from non-damaged DNA, yielding a characteristic ‘comet tail’ shape; however, some breakage is not caused by oxidative damage to DNA

Abbreviations: ELISA, enzyme-linked immunosorbent assay; GC-MS gas chromatography–mass spectrometry; HNE, 4-hydroxy-2-nonenal; HPLC, high-performance liquid chromatography; MDA, malondialdehyde.

**Table 2:** Summary of methods that can be used to determine antioxidant defences in conservation studies, from biological matrices that do not require terminal sampling

Method	Biological matrix	Description
Non-enzymatic antioxidant capacity	Serum, plasma, red blood cells, egg (both yolk and albumen), urine, seminal plasma, tissue biopsy, faeces, colostrum/milk	Many colorimetric assays (OXY-adsorbent test, FRAP test, ORAC test, TEAC) are available to quantify the *in vitro* reaction between antioxidants and a given pro-oxidant. Some assays (FRAP and TEAC) suffer from the fact that they are mostly sensitive to certain antioxidants, such as uric acid
Antioxidant defences of red blood cell membranes (KRL test)	Whole blood	It measures the time needed to haemolyse 50% of red blood cells during *in vitro* exposure to a pro-oxidant; it provides a quantification of antioxidant defences that occur on the cell membranes of red blood cells, but also includes the contribution of non-enzymatic antioxidants that occur in the liquid component of whole blood
Antioxidant enzymes	Red blood cells, immune cells, seminal plasma, tissue biopsy, colostrum/milk	Colorimetric assays determine the *in vitro* activity of enzymes, which is dependent on their concentration. Commonly measured antioxidant enzymes are superoxide dismutase, glutathione peroxidase, catalase, glutathione reductase and glutathione-*S*-transferase
Thiols	Serum, plasma, red blood cells, immune cells, tissue biopsy	Thiols are molecules that have a carbon-bonded sulfhydryl (–C–SH or –R–SH) group; they can be of protein or non-protein origin. They are very sensitive to environmental stressors that induce an increase in production of reactive oxygen species. Colorimetric assays are available to determine their concentration. Quantification of the reduced (GSH) and oxidized (GSSG) forms of glutathione provides a good measurment of the redox equilibrium. Oxidation of protein thiols is increasingly recognized to govern ageing mechanisms and cell homeostasis

**Figure 2:**
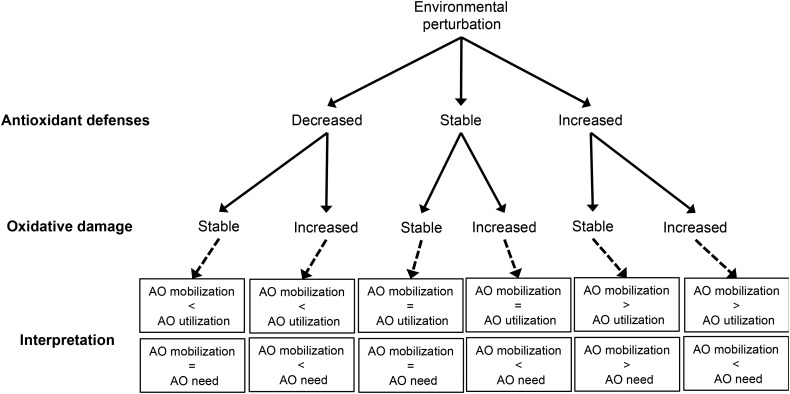
Theoretical scenarios regarding alteration of the oxidative balance [antioxidant defences (AO) and oxidative damage] of animals subjected to an environmental disruption. Mobilization is considered with a broad meaning, i.e. the mobilization of exogenous antioxidants and/or the up-regulation of endogenous antioxidants. Note that oxidative damage may also decrease following environmental disruptions, presumably because of a reduction in metabolism associated with low body reserves or because of increased mitochondrial uncoupling. Given that this scenario has been reported rarely following environmental disruption (e.g. Beaulieu *et al.*, 2013) and in order to keep the figure as clear as possible, it is not represented here.

The dual-faceted aspect of oxidative stress (antioxidant defences vs. oxidative damage) makes it a unique physiological marker to assess the protective response that animals are able to mount in order to cope with environmental stressors, as well as the deleterious effects they undergo when facing adverse conditions. For instance, an elevation of antioxidant defences coupled with stable levels of oxidative damage suggests that animals are able to protect themselves against the new oxidative conditions they face. In contrast, decreased antioxidant defences coupled with increased levels of oxidative damage suggest that they are unable to respond to new conditions, leading to an exhaustion of antioxidant defences and a rise in oxidative damage. Obviously, any intermediate situation between these two extremes is possible (Fig. 2). We should not lose sight of the fact that elevating antioxidant defences might not be cost free if it requires the diversion of energy or nutrients from other functions. For example, using dietary antioxidants (which are also renowned immunostimulants) for mitigating oxidative stress would mean that they are not longer available to sustain the immune response. Likewise, decreased antioxidant defences might also indicate that the organism is favouring investment in other functions that impact more on evolutionary fitness in those specific circumstances.

Another appealing aspect of markers of oxidative stress for conservationists is their universality. Indeed, while measurements of glucocorticoids are confined to vertebrates (corticosterone or cortisol according to the taxon considered), the measurement of markers of oxidative stress can, in principle, be applied to any species with aerobic metabolism and even to anaerobic organisms exposed to oxygen (Imlay, 2003; Costantini, 2014), making markers of oxidative status applicable to a wide range of organisms, from bacteria to humans.

Finally and importantly for conservation purposes, markers of oxidative status cannot only be used to describe the individual physiological status, but also to make predictions about individual perspectives of survival or reproduction. For instance, it has been found that markers of oxidative status can predict recruitment, fledging success or survival in different bird species (Saino *et al.*, 2011; Noguera *et al.*, 2012; Losdat *et al.*, 2013), although such a link between oxidative status and survival does not always emerge. For instance, the link between markers of oxidative status and proxy variables of survival (recruitment probability of young, probability to survive until the next year) in free-living birds is found in 41.7% of studies (Costantini, 2014). Discrepancies among studies therefore show that some cautiousness in interpretation is needed in the early stages of application of markers of oxidative status in wildlife management programmes.

Despite the clear advantages associated with the measurement of markers of oxidative status to assess how animals cope with new environmental conditions (e.g. fitness relevance, ‘protection/damage’ dual aspect, universality, predictive component), they have only been reported recently and anecdotally in conservation studies if compared with the vast conservation literature on glucocorticoids and immune parameters (Fig. 1). As such, markers of oxidative status can reasonably be considered as missing tools in conservation physiology.

## *A posteriori* effects of environmental disruptions on markers of oxidative status

**Figure 3:**
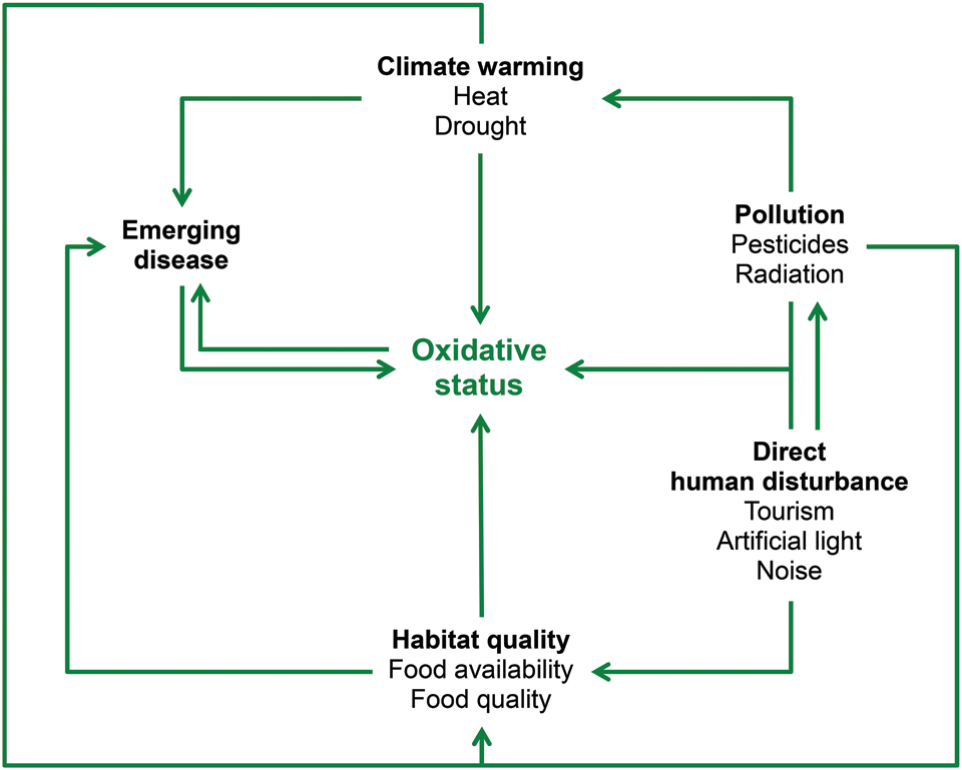
Schematic diagram showing the environmental disruptions due to human activities that are likely to affect the oxidative balance of animals. An arrow between two factors indicates a causal relationship.

Human activities can impinge on individual oxidative status in a myriad of ways (Fig. 3). Conservationists may therefore be interested in using markers of oxidative status to assess the effects of human activities on natural animal populations. However, a prerequisite for such an approach is to identify these anthropogenic factors and envision their potential effects on the oxidative status of animals.

### Direct human disturbance

Irrespective of human activities, the direct presence of humans in the vicinity of wild animals can by itself affect their oxidative status through its anxiogenic effects (Bouayed *et al.*, 2009). Such effects may occur if humans are considered by animals as potential new predators against which they are not well armoured (Frid and Dill, 2002). For example, it has been found that increased predation risk can influence the oxidative status in many species and always in a similar way, i.e. a decrease in antioxidant defences coupled with an increase in oxidative damage. This is the case in scallops (*Argopecten ventricosus*) exposed to blue crabs (*Callinectes sapidus*; Guerra *et al.*, 2013), in larvae of damselflies (*Enallagma cyathigerum*) exposed to visual or chemical cues from larvae of dragonflies (*Anax* spp.; Janssens and Stoks, 2013) and in female song sparrows (*Melospiza melodia*) exposed to frequent experimental nest predation (Travers *et al.*, 2010). Given that ecotourism forces animals to be exposed to human presence, it is likely to generate anxiety, thereby potentially altering their oxidative status. Despite the large number of studies examining the effects of tourism on wildlife (Davenport and Davenport, 2006; Item *et al.*, 2008; Knapp *et al.*, 2013), to our knowledge only one study has directly examined the effects of tourism on markers of oxidative status. In this study, the authors found that southern stingrays (*Dasyatis americana*) from the Cayman Islands occupying habitats with high tourist pressure had decreased levels of serum antioxidant capacity and increased levels of serum oxidative damage (Semeniuk *et al.*, 2009), similar to what has been found in studies on animals exposed to predation risk.

### Urbanization

Regarding the impact of human presence on the oxidative status of animals, the effects of urbanization have received more attention than tourism. Urbanization is characterized by a multitude of modifications of the natural habitat, among which changes in light and sound levels are likely to have major impacts on the oxidative status of animals. Given that artificial light disrupts the organizational structure of the environment, it is likely to alter the oxidative status of animals that inhabit urban areas (Navara and Nelson, 2007). For instance, rats constantly exposed to light exhibit decreased antioxidant enzymatic activity and increased oxidative damage (Baydas̨ *et al.*, 2001; Cruz *et al.*, 2003). These effects are likely to be mediated by the suppressive effect of light on the nocturnal secretion of melatonin (Dominoni *et al.*, 2013), a hormone that has powerful antioxidant properties but which is also able to stimulate the synthesis of other endogenous antioxidants (Tan *et al.*, 2010). Accordingly, the administration of melatonin to rats constantly exposed to light restores their oxidative status to normal levels (Baydas̨ *et al.*, 2001; Cruz *et al.*, 2003). The effects of artificial light on the oxidative status of animals are likely to influence a large number of animal species, because light pollution can be found on ∼20% of the terrestrial surface (Longcore and Rich, 2004). Importantly, these effects are likely to be worsened by other factors associated with urbanization, such as anthropogenic noise. For instance, living close to a road exposes animals to artificial light but also to the noise of traffic, which can reach 60–80 dB (Theebe, 2004). Experimental work has shown that exposing rats for 4 h/day (for 1–30 days) to 100 dB significantly increases the levels of both antioxidant enzymes and oxidative damage in their brain (Samson *et al.*, 2005). As with the effects of light pollution, the effects of anthropogenic noise (mostly due to traffic) on the oxidative status are likely to impact on a wide variety of animals. For instance, in 1995, 20% of the total land of the conterminous USA was within 127 m of a road, and 83% within 1061 m of a road (Riitters and Wickham, 2003). Importantly, the deleterious effects of roads on the oxidative status can be aggravated due to the fumes and particles that are produced by traffic. These effects have been well studied in view of human health issues, with low air quality increasing oxidative damage (Li *et al.*, 2002; Møller and Loft, 2010), but to our knowledge, they have not yet been considered in conservation or wildlife management studies.

### Pollution

In comparison to light or noise pollution, the effects of contamination of natural habitats with persistent organic pollutants, pesticides, heavy metals or ionizing radiation can be more persistent (Carere *et al.*, 2010; Isaksson, 2010; Walker *et al.*, 2012). The mode of action of some pesticides, such as bipyridyl herbicides (paraquat and diquat) that are used worldwide for agricultural purposes, is precisely to alter the oxidative status of plants in order to eliminate them (Jones and Vale, 2000). If animals are exposed to these pesticides, they in turn can undergo a similar disruption of their oxidative status, which may be fatal to them (van Oers *et al.*, 2005). Importantly, the pro-oxidant effects of pesticides are not restricted to bipyridyl herbicides, but they represent the central mechanism that unifies the toxicity of most pesticides (Banerjee *et al.*, 2001). Likewise, a disruption of the oxidative status appears to represent the unifying mechanism of the toxicity related to metal contamination (Koivula and Eeva, 2010). On the one hand, redox-active metals, such as copper, iron, vanadium or zinc, increase the production of ROS, while on the other hand, redox-inactive metals, such as cadmium, lead, mercury and nickel, primarily deplete important endogenous antioxidants, such as glutathione (Ercal *et al.*, 2001). Contrary to pesticides, metals are not easily degraded into less toxic compounds; hence, their action is likely to be highly pernicious and long lasting because of their persistence in both the environment and the organism. Moreover, metals, such as mercury, can accumulate in food webs (Luoma and Rainbow, 2005), so that top predators represent the most probable organisms in which the effects of metal pollution on markers of oxidative status may be observed.

Ionizing radiation, which can also affect the oxidative status of animals by directly elevating ROS production (Riley, 1994), can have even more long-lasting effects than metals. For instance, more than 20 years after Chernobyl disaster, barn swallows (*Hirundo rustica*) from this region still show signs of contamination, such as high levels of plasma oxidative damage (Bonisoli-Alquati *et al.*, 2010) or low levels of carotenoids and vitamins in eggs (Møller *et al.*, 2005), in comparison to barn swallows from control areas. Importantly, our understanding of the effects of radioactive contamination on the oxidative status of animals is, for the moment, very limited and lacks perspective, because such contamination is expected to persist in the environment for thousands of years (Christodouleas *et al.*, 2011).

### Climate change

Climate change, and climate warming in particular, represents another critical consequence of human activities that can affect the oxidative status of animals. Indeed, high temperatures increase metabolism, passively in ectotherms, because of elevated basal metabolism in hot conditions, or actively in endotherms to eliminate extra heat and maintain inner temperature constant despite high environmental temperatures (Blagojević, 2007; Angilletta, 2009; Dillon *et al.*, 2010). As such, exposure to extreme temperatures (e.g. during heat waves) might alter the oxidative status of ecto- and endotherms by increasing their production of ROS (Lin *et al.*, 2008; Tumminello and Fuller-Espie, 2013). If animals are not able to compensate fully for an increase in ROS production by mobilizing antioxidants, exposure to high temperature is expected to result in increased oxidative damage. For instance, heat-stressed lace bugs (*Corythucha ciliata*) can only protect themselves partly against heat-induced oxidative stress, as illustrated by an increase in antioxidant enzymes accompanied by an increase in oxidative damage levels (Ju *et al.*, 2014). In contrast, Japanese quails (*Coturnix coturnix japonica*) do not appear to be able to protect themselves against heat-induced oxidative stress at all, as suggested by a decrease in antioxidant enzymes and a concomitant increase in oxidative damage (Sahin *et al.*, 2012). Importantly, the intensity and the duration of thermal stress are likely to determine the speed, the intensity and the persistence of the response of the organism, which may explain response differences between studies.

Droughts represent another consequence of climate warming that may affect the oxidative status of animals (Dai, 2012). A decrease in access to water, and thereby an increase in risk of dehydration for animals, have been associated with changes in oxidative status, leading to the suggestion that ‘oxidative damage [is] one of the most deleterious effects of water depletion’ (França *et al.*, 2007). Intuitively, one may suppose that this is more problematic in animals, such as amphibians, that are highly dependent on water and are therefore very sensitive to dehydration. However, some evidence suggests that amphibians, possibly because they are frequently exposed to threats of dehydration, are fully able to activate their antioxidant machinery during dehydration to avoid high levels of oxidative damage. This has been illustrated in leopard frogs (*Rana pipiens*), which show increased antioxidant enzymes while dehydrated, thereby maintaining oxidative damage stable (Hermes-Lima and Storey, 1998). Surprisingly, a study conducted on a species adapted to dry environments, the one-humped camel (*Camelus dromaderius*), found different results. Indeed, contrary to amphibians, camels exposed to 20 days of water deprivation are only partly able to compensate for the effects of dehydration on oxidative status, as suggested by increased levels of glutathione (an endogenously synthesized antioxidant) associated with an increase in oxidative damage (Ali *et al.*, 2013).

### Food availability

In addition to their direct effects on the oxidative status of animals, human disturbance, urbanization, pollution and climate change can also have indirect consequences on the oxidative status of animals through their effects on the availability of food resources (e.g. decreased food abundance, reduced access to food resources, mismatch between food production and consumers' requirements; Durant *et al.*, 2007). For instance, climate warming around the Antarctic Peninsula is thought to be responsible for the depletion of Antarctic krill (*Euphausia superba*) in the Southern ocean (Atkinson *et al.*, 2004). As a response, krill consumers (whales, seals and seabirds) may have to intensify their foraging effort to fulfil their feeding requirements, which may result in higher ROS production. Simultaneously, they would need to increase their antioxidant defences to keep oxidative damage stable (Beaulieu *et al.*, 2011). In contrast, Adélie penguins (*Pygoscelis adeliae*) from the Antarctic Peninsula show both lower plasma antioxidant capacity and lower levels of oxidative damage than Adélie penguins from other regions (Beaulieu *et al.*, 2013). These results closely mirror those obtained in captive mallards (*Anas platyrhynchos*) after fasting for several days (Geiger *et al.*, 2012). In both cases, reduced antioxidant defences coupled with reduced oxidative damage may reflect a reduction of the metabolism of birds, as a response to low energy body reserves due to low food availability, as well as an increased uncoupling of mitochondria in starving birds that enhances mitochondrial efficiency and lipid utilization (Evock-Clover *et al.*, 2002; Seifert *et al.*, 2008). In contrast, Seychelles warblers (*Acrocephalus sechellensis*) inhabiting poor-quality territories (with low insect density) show stable plasma antioxidant capacity but increased oxidative damage (van de Crommenacker *et al.*, 2011). These higher levels of oxidative damage suggest that they may still be able to intensify their foraging effort, although they do not mobilize antioxidant defences concomitantly. In common dentex (*Dentex dentex*), prolonged starvation is associated with increased oxidative damage together with an increase in the activity of antioxidant enzymes (Morales *et al.*, 2004). This suggests that starvation represents a pro-oxidant stressor for this fish, against which it is only able to respond partly.

Human activities may also affect the availability of food resources by directly transforming the structure and the composition of the environment. Importantly, more than 50% of the land surface of the Earth has been modified by human activities (e.g. agriculture, industry, forestry; Hooke *et al.*, 2012). The resulting reorganization and impoverishment of lands in terms of resource diversity available for animals inhabiting human-altered areas is likely to affect their oxidative status. For instance, common scale-backed antbirds (*Willisornis poecilinotus*) inhabiting forests with frequent natural tree-fall gap openings in the canopy have higher oxidative damage than birds inhabiting forests with undisturbed canopy (Gomes *et al.*, 2014), thereby suggesting that logging may disrupt the oxidative status of forest animals. Likewise, honey bees (*Apis mellifera*) transferred to greenhouses where only strawberries or eggplants occur show a down-regulation of antioxidant system genes coupled to an increase in oxidative damage compared with bees from locations with greater plant diversity (Morimoto *et al.*, 2011). This pattern may be observed if habitat impoverishment forces bees to increase their foraging effort or if it deprives them of plant secondary compounds acting as antioxidants (polyphenols, vitamin C, vitamin E and carotenoids; Beaulieu and Schaefer, 2013). These compounds can indeed act directly as antioxidants in the organism or can stimulate the expression of antioxidant enzymes by consumers (Moskaug *et al.*, 2005; Spanier *et al.*, 2009; Yeh *et al.*, 2009). Consequently, if animals relying on dietary antioxidants can no longer find such resources in their environment, they are likely to show altered oxidative status, characterized by low antioxidant defences and potentially high oxidative damage. For example, a low consumption of krill by Adélie penguins around the Antarctic Peninsula may also explain their low antioxidant defences (Beaulieu *et al.*, 2013), because krill rich in astaxanthin (a carotenoid) may contribute to their overall antioxidant defences (Beaulieu *et al.*, 2010). Likewise, feeding on human wastes (e.g. fishery wastes) or food provided by humans (e.g. food provided to birds in winter) may also alter the antioxidant intake of animals, if such a strategy diverts their feeding behaviour from the usual antioxidant-rich resources. This may explain the negative effects of such a feeding strategy on bird reproduction (Grémillet *et al.*, 2008; Plummer *et al.*, 2013a). The recently formulated hypothesis that food of human origin deprives animals of dietary antioxidants would deserve closer attention in future conservation studies (Plummer *et al.*, 2013a, b).

### Outbreaks of infection

The redistribution of infectious diseases at the surface of the planet represents another major side-effect of human activities that animals have to deal with. The emergence of new diseases may be due to climate change (Lafferty, 2009; Altizer *et al.*, 2013), but also to the transportation of contaminated goods, animals and humans at a high rate and on a global scale (Saker *et al.*, 2004). Such a situation greatly facilitates the transmission of diseases to animals through different new vectors (e.g. humans *per se*, livestock or pets, human food, waste water). This high exposure to new infectious diseases may be reflected by changes in the oxidative status of infected animals. This is because an organism first responds to infection through the production of non-specific toxic compounds (such as ROS) to eliminate infectious agents. Importantly, because of their non-specificity, ROS are also toxic to the host's tissues (Sorci and Faivre, 2009). This implies the existence of a cost/benefit ratio associated with the production of ROS following infection. Therefore, infection must be followed by a properly timed and transient up-regulation of ROS production coupled with a down-regulation of antioxidant defences, resulting in a temporary increase in oxidative damage. Accordingly, Eurasian kestrels (*Falco tinnunculus*) that have been experimentally immunostimulated exhibit lower plasma antioxidant capacity and increased oxidative damage (Costantini and Dell'Omo, 2006). Importantly, the effects of infection on the oxidative status of animals may be noticeable only when infection is coupled to other pro-oxidant conditions. For instance, Seychelles warblers infected with malaria show comparable plasma antioxidant capacity and oxidative damage to non-infected birds during the pre-nesting period and during incubation. In contrast, during the energetically demanding period of chick provisioning, levels of oxidative damage increase in infected parents, while they do not do so in non-infected parents (van de Crommenacker *et al.*, 2012).

Environmentally induced oxidative stress may also favour outbreaks of viral infections in animal populations. For example, infection with herpes virus can be facilitated by a cell state of oxidative stress, which can be inhibited by dietary antioxidants, such vitamin E or resveratrol, thereby accelerating lesion healing and recovery (Faith *et al.*, 2006; De Luca *et al.*, 2012). For instance, resveratrol was found *in vitro* to inhibit the replication of the virus responsible for duck enteritis (an acute, contagious herpes virus infection of ducks, geese and swans) in a dose-dependent manner (Xu *et al.*, 2013). This viral inhibition was not attributed to direct inactivation or inhibition of virus attachment to the host cells, but to the inhibition of viral multiplication in host cells (Xu *et al.*, 2013). Other viruses can also exploit a state of slight oxidative stress of the cell to increase their replication. For example, deficiency in dietary antioxidants (vitamin E) and cofactors of glutathione peroxidase (selenium) can cause transition of avirulent to virulent or virulent to extremely virulent mutation of coxsackievirus in mice (Beck, 1997).

### Interactions and synergies

We have provided here a list of anthropogenic threats that are able to impact on the oxidative status of wild animals: human presence, tourism, light pollution, noise, fumes, pesticides, metal, radiation, climate warming, drought, food availability and emerging diseases. These pro-oxidant factors may therefore be of interest for conservationists concerned about the physiological response of animals to new environmental conditions. However, so far each of these factors has been examined separately, and we still lack a clear view of how these factors may interact with each other. Considering these factors in combination may, in fact, better reflect the real impact of environmental disruptions on oxidative status, because animals are not likely to experience each of these pro-oxidant factors independently (Fig. 3). For instance, tourism is not only associated with the anxiogenic effects of human presence, but it is also associated with other side-effects, such as exposure to unnatural food resources and to diseases of anthropogenic origin. Likewise, animals living close to roads are simultaneously exposed to the effects of light, noise and car fumes. How these different environmental stressors act simultaneously (and potentially in synergy) on the oxidative status of animals remains to be determined.

## *A priori* assessment of biomarkers of oxidative status in conservation programmes

Reintroductions and translocations are frequently used methods in conservation biology. They are important, for example, to increase gene flow, to enlarge populations or to repopulate areas where a certain species has become extinct. The success of reintroduction and translocation programmes can, however, be particularly low because animals frequently die soon after release, presumably because of the stress related to such conservation actions (Teixeira *et al.*, 2007). What is lacking to date is a clear understanding of the mechanisms that underlie the detrimental effects of stress on individual survival. As shown in the examples listed in the previous section, oxidative stress can certainly be one important mechanism involved, given its links with stress hormones (Costantini *et al.*, 2011a), fertility (Helfenstein *et al.*, 2010) or survival (Saino *et al.*, 2011). In this regard, it is very important to highlight that there are differences in the resistance to oxidative stress induced by environmental perturbations not only for species, but also for individuals and conspecific populations. This suggests that biomarkers of oxidative status could also be used to assess *a priori* whether the physiological and genetic attributes of an individual to be reintroduced or translocated are compatible with those of the target area or population.

### Individual variation

Conspecific individuals of the same sex or age often differ from each other in clusters of behaviour and underlying physiology, even in standardized conditions. While some individuals tend to be highly responsive to stimuli, others are unresponsive and show routine-like behaviours (Carere and Maestripieri, 2013). Inter-individual variations in behaviours to cope with stressful episodes can be modelled along an axis polarized at the two extremes by proactive and reactive responses (Koolhaas *et al.*, 2010). Proactive individuals are generally bold, superficial explorers, active and aggressive. In contrast, reactive individuals are less active and aggressive, but explore novel environments thoroughly. Differences in coping styles have been considered to reflect consistent behavioural differences among individuals, and may be referred to as personalities (Carere and Maestripieri, 2013). Personalities also differ in metabolism (Careau *et al.*, 2008) or glucocorticoid response (Carere *et al.*, 2003). It is generally thought that variation among individuals is maintained because, while certain behavioural phenotypes do better than others in certain environmental conditions, an opposite pattern can emerge in different conditions (Carere and Maestripieri, 2013). Assessment of physiological end-points might therefore be useful in evaluating how an individual responds to environmental stimuli and in predicting consequences for individual fitness.

In recent years, research on various vertebrate species has shown that personality types can differ in oxidative profile. For example, in a study on wild Alpine marmots (*Marmota marmota*), it was found that different individual coping styles may be associated with differences in pre-restraint or acute stress-induced blood oxidative status in a natural setting (Costantini *et al.*, 2012). Marmots with a more proactive coping style had higher baseline levels of plasma non-enzymatic antioxidants and experienced higher oxidative damage when exposed to an acute stressor (i.e. a 30 min restraint and an open-field test).

Links between personality and oxidative status were also found in birds and reptiles using wild-caught animals tested in captive conditions. A report on greenfinches (*Carduelis chloris*) showed that neophobic individuals had lower plasma non-enzymatic antioxidant capacity and higher plasma oxidative damage than neophilic ones; and that fast-exploring individuals had higher plasma non-enzymatic antioxidant capacity and lower plasma oxidative damage than slow-exploring ones (Herborn *et al.*, 2011). In a study on the White's skink (*Egernia whitii*), Isaksson *et al.* (2011) found that more aggressive males had higher plasma non-enzymatic antioxidant capacity but not higher oxidative damage than less aggressive males. Overall, these studies show that biomarkers of oxidative status may prove a valuable tool to assess the individual's stress responsiveness, and thereby their ability to cope with new environmental conditions following habitat disruption or reintroduction into new habitats. Although measurements of stress hormones may also provide quantification of individual responsiveness to translocation or reintroduction, biomarkers of oxidative status offer additional value in that they may provide further information on fitness consequences, given the links between oxidative stress and fertility (Helfenstein *et al.*, 2010), immune status (Costantini and Dell'Omo, 2006), susceptibility to pathogens (Mougeot *et al.*, 2009) or survival prospects (Saino *et al.*, 2011). For instance, markers of oxidative damage or antioxidant status in youth may predict survival in adulthood, as well as recruitment probability (Noguera *et al.*, 2012; Losdat *et al.*, 2013). Hence, animal-breeding programmes, which are aimed at reintroducing captive-born individuals into natural habitats, should control captivity conditions in which animals live (to limit oxidative stress) and select individuals with an oxidative status that best fits with the environment in which they will be reintroduced. Such an approach might ensure high survival prospects and long-term reintroduction success.

### Population variation

Various reports have shown that conspecific populations can differ significantly in their sensitivity to oxidative stress. This suggests that it may be crucial to assess the oxidative status of the host population before introducing individuals into that population. For example, in an environment that is particularly challenging, selection may favour individuals highly resistant to oxidative stress. The introduction of individuals that are poorly protected against oxidative stress into that population would be pointless because they would be counter-selected. Conversely, introduction of a highly resistant individual into a population where there is not much need to be so strong may not be optimal, because of the costs associated with the maintenance of a long-lasting phenotype in the absence of challenging conditions. We also need to bear in mind that the resistance of an organism to oxidative stress has a genetic basis. Hence, the introduction of genes that are not compatible with the genetic background of the target population should be avoided.

Examples of inter-population differences in terms of oxidative status have been provided across different animal taxa, from invertebrates to vertebrates. These differences may be of environmental origin. For example, Adélie penguins from the Antarctic Peninsula have lower non-enzymatic antioxidant capacity and lower oxidative damage than conspecific penguins from East Antarctica, presumably because of a lower contribution of krill to their diet (Beaulieu *et al.*, 2013). Likewise, populations of Galápagos land iguanas (*Conolophus subcristatus*) from various islands of the archipelago show differences in plasma oxidative damage, plasma non-enzymatic antioxidant capacity or plasma carotenoids (Costantini *et al.*, 2005, 2009). In particular, results of these studies suggested that reproductive activity, food availability and local pressures related to human activity might have contributed to explaining the variation in oxidative profile, because mating seasons, food abundance or human impact differed among islands (Snell *et al.*, 1984). Many differences in stress physiology were also found between urban and rural animal populations, suggesting the importance of human activities as a new selective agent (*Turdus merula* in Partecke *et al.*, 2006; *Passer domesticus* in Herrera-Dueñas *et al.*, 2014; reviewed by Sol *et al.*, 2013). A comparative study on many bird species found that rural avian populations had higher concentrations of vitamin E and carotenoids in liver than conspecific urban populations (Møller *et al.*, 2010). Differences between conspecific populations were accounted for by the quality of the diet (Møller *et al.*, 2010). Compared with rural populations, urban populations of common side-blotched lizards (*Uta stansburiana*) showed higher levels of stress hormones, which were associated with higher oxidative damage and suppressed immunity. Moreover, urban populations had higher reproductive output and decreased survival (Lucas and French, 2012). Overall, this evidence highlights how important it is to assess oxidative status when planning reintroduction programmes in rural populations or when interpreting variation among populations in the response to urbanization. The extent to which a given marker of oxidative status will succeed in predicting the success of the reintroduction programme will be likely to vary across species and environmental contexts, depending, for example, on the risk of extrinsic mortality (e.g. predation) or the intensity of the fluctuations in environmental abiotic factors (e.g. weather conditions, impact of human activities).

Inter-population differences in markers of oxidative status can also be of genetic origin. For instance, Robert and Bronikowski (2010) found that life-history differences between ecotypes of the western terrestrial garter snake (*Thamnophis elegans*) are also translated at the physiological level. Compared with the short-lived phenotype, offspring snakes from the long-lived ecotype consumed equal amounts of oxygen, produced more energy for a given amount of consumed oxygen, had lower mitochondrial production of ROS and had DNA in red blood cells that was damaged more readily but was also repaired more efficiently. Divergences between ecotypes in the mechanisms regulating oxidative status are not limited to vertebrates, but have a general applicability. For example, Philipp *et al.* (2012) found that oxidative status differed between short- and long-lived populations of the bivalve *Arctica islandica* (living <50 years and until 410 years, respectively).

The importance of considering markers of oxidative status in reintroduction and translocation programmes is further strengthened by the fact that genetic inbreeding (increase in homozygosity) may make individuals more sensitive to damage. Inbreeding depression results in reduced evolutionary fitness (e.g. Lynch and Walsh, 1998) that makes organisms less resistant to environmental stress, including oxidative stress (e.g. Pedersen *et al.*, 2008; Okada *et al.*, 2011). For example, levels of testicular stress were significantly elevated in inbred male fruitflies, which might jeopardize male fertility (Okada *et al.*, 2011).

## Methods and interpretation

Theoretically, markers of oxidative status can be measured in any biological samples. Various methods have been described in several recent publications (Monaghan *et al.*, 2009; Hõrak and Cohen, 2010; Costantini, 2011). Hence, in the remainder of this article, we will emphasize only methodological aspects that may be important in conservation studies.

In species of conservation concern and in reintroduction programmes, physiological measurements necessarily need to be restricted to biological matrices for which sampling is as non-invasive as possible and which does not lead to the death of animals. We have summarized in Tables 1 and 2 the methods that can be applied to biological matrices that do not require terminal sampling. For instance, the collection of blood in vertebrates, sperm in birds (Helfenstein *et al.*, 2010), haemolymph in large arthropods (Criscuolo *et al.*, 2010) or fat or muscle biopsies in large marine mammals (Hunt *et al.*, 2013) can be done relatively easily, without putting the life of animals at risk. Importantly, tissue sampling inevitably requires animals to be tracked, trapped, captured, restrained (physically or through anaesthesia) and potentially transported. If possible, investigators should ensure that such an approach does not interfere excessively with measurements of markers of oxidative status. For instance, anaesthesia can impact on markers of oxidative status, as demonstrated in human and veterinary medicine (Allaouiche *et al.*, 2001; Simeonova *et al.*, 2004). Transportation has also been described as decreasing antioxidant defences and elevating oxidative damage in domestic goats (*Capra hircus*; Nwunuji *et al.*, 2014), whereas it does not have any effects in wild European badgers (*Meles meles*; Montes *et al.*, 2011). Consequently, when possible, the extent to which the sampling of wild animals might affect markers of oxidative status needs to be determined.

Measurement of markers of oxidative status in a single biological matrix may not be optimal, because it may not necessarily reflect the oxidative status of the whole organism or the oxidative status of tissues that mostly impinge on fitness. For example, a combination of matrices such as blood (both plasma and red blood cells), urine (for those species that urinate), tissue biopsies and sperm might provide a good picture of oxidative stress to both soma and germ cells (see Tables 1 and 2). A first pre-requisite for conservationists is therefore to examine how the markers of oxidative status that they wish to use relate to fitness components in their biological model. Such an approach would allow them to confirm that a given marker of oxidative status provides information that is ecologically relevant and may therefore be useful in conservation studies.

Although differential sensitivity of markers of oxidative status can be expected, two meta-analyses showed that the sensitivity to strong chronic stressors did not differ significantly. Isaksson (2010) found that the effects of pollution were fairly similar between biomarkers of oxidative damage (malondialdehyde and thiobarbituric acid reactive substances) and some antioxidants (glutathione, glutathione peroxidase and glutathione-*S*-transferase). Likewise, Costantini *et al.* (2011a) found that various biomarkers (e.g. thiobarbituric acid reactive substances, glutathione, catalase, superoxide dismutase, glutathione peroxidase) did not differ significantly in susceptibility to physiological stress induced by glucocorticoids.

It is important to emphasize here that, as in any other studies dealing with oxidative status, conservation studies should assess both constituents of oxidative status in order to be able to interpret their results: (i) antioxidant defences, reflecting the protective response that animals are able to mount when facing adverse conditions; and (ii) oxidative damage, reflecting the deleterious effects they undergo (Fig. 2). However, even after measuring both antioxidant defences and oxidative damage, the information they provide may still not be univocal in terms of costs and benefits for animal populations. For instance, low antioxidant defences coupled with high levels of oxidative damage in breeding animals are likely to reflect a high investment in reproduction and low investment in self-maintenance. This may be beneficial in terms of population health for short-lived animals but deleterious for long-lived animals. Consequently, conservationists need to have a pre-existing knowledge of the following factors: (i) which environmental conditions are optimal for animal populations; (ii) how variations of environmental conditions affect them in terms of fitness and population health; and (iii) how markers of oxidative status reflect these variations. This suggests that it is necessary to examine the full sequence of events ‘environmental disruption → oxidative status → fitness effects’ and not only parts of it (e.g. only ‘environmental disruption → oxidative status’ classically reported in studies in oxidative stress ecology as described in the section above entitled ‘*A posteriori* effects of environmental disruptions on markers of oxidative status’). Towards this end, it is important to analyse the variation of markers of oxidative status in populations subject to different environmental conditions and characterized by different outputs in terms of body condition, breeding success, survival or demographic trends. This approach can first be conducted in populations geographically separated and that experience different environmental conditions. For instance, we previously found that declining populations of *Pygoscelis* penguins experiencing climate warming exhibit lower antioxidant defences than other populations, and that demographic trends and antioxidant defences were so tightly bound that antioxidant defences might be used by conservationists as an index to estimate the population health of populations of unknown demographic status (Beaulieu *et al.*, 2013).

Prior knowledge of the oxidative status of individuals within a potential host population may also be useful for conservation programmes aiming to reintroduce animals into new habitats. As illustrated in the above paragraphs, it is essential for reintroduction programmes to maximize the chances of survival of the individuals being reintroduced. Towards this end, *a priori* assessments of the oxidative status of the individuals to reintroduce and of the host population may increase the chances of success, because some individuals are likely to be a better match for the oxidative status phenotype of the host population.

It is important to emphasize that, within a species, markers of oxidative status do not fluctuate only because of exposure to stress but also because of natural variation throughout life. For instance, oxidative damage can increase during growth, reproduction or senescence (Finkel and Holbrook, 2000; Alonso-Álvarez *et al.*, 2004; Stier *et al.*, 2012). It is therefore imperative to differentiate variations in markers of oxidative status that are due to natural events from those due to conservation threats. A precise knowledge of the life stage of the sampled animals is thus essential. It may also be important to have information about life stages because this would provide the possibility of identifying the life stage at which oxidative status is most susceptible to environmental disruptions. Furthermore, most animals included in ecophysiological studies are sampled during the breeding season, when they are accessible to investigators. Therefore, results obtained during this season are highly influenced by reproduction, and may reflect how breeding animals trade self-maintenance against current reproduction. As short-lived parents are expected to allocate more resources into current reproduction than into self-maintenance (Stearns, 1989), the effects of environmental disruptions on oxidative status should be more pronounced in parents than in offspring. In contrast, in long-lived species, where parents should favour self-maintenance at the expense of current reproduction when resources are limited, the effects of environmental disruptions on oxidative status should be more severe in offspring than in their parents. These theoretical examples show that it is important for conservationists to consider life-history traits in order to be able to interpret variation in oxidative status due to environmental disruption.

As stated in the aforementioned paragraphs, quantification of the variation of markers of oxidative status in relation to individual life stages and attributes and to environmental conditions would be a first step to define reference values for the population or species target. Another relevant step would be to assess whether certain diseases or parasitic infections typical of the species under study result in increased levels of oxidative damage or reductions in antioxidant defences. Such an assessment would provide relevant information about the diagnostic value of a given marker of oxidative status. Another important preliminary step in the application of oxidative status markers in wildlife management would be to complement baseline values with reference values collected from captive or wild animals (when possible) subjected to different stressful conditions. This would provide information about stress-induced values of markers of oxidative status and about the magnitude (e.g. maximum peak) and the timing of these changes (e.g. acute vs. chronic, time lag in the response).

**Figure 4:**
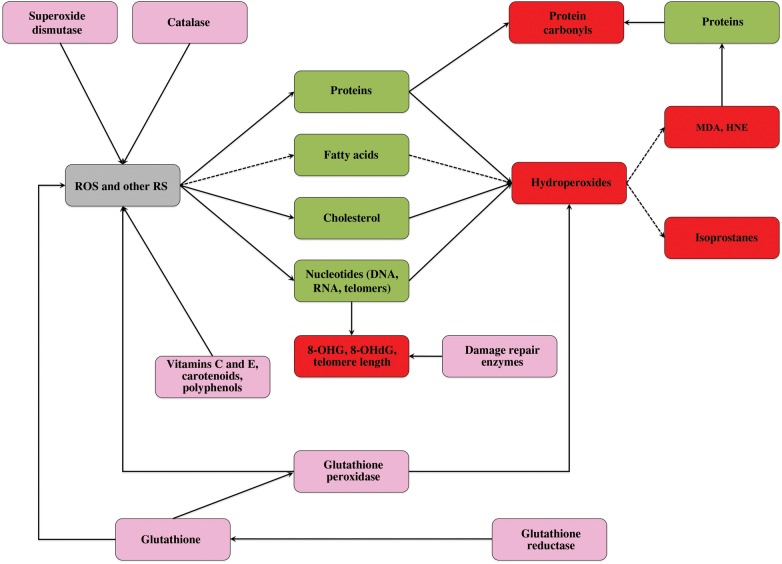
Schematic and simplified representation of molecular interactions among some of the most common biomarkers of oxidative damage and antioxidant defences. Oxidation of fatty acids gives rise to early derivatives of oxidative damage called hydroperoxides; these are precursors of end-products of lipid peroxidation, such as malondialdehyde (MDA), 4-hydroxy-2-nonenal (HNE) and isoprostanes. In turn, MDA and HNE can form adducts with proteins, generating protein carbonyls. Protein carbonyls can also be generated by direct oxidation of proteins caused by reactive species (RS). Note that other peroxidation pathways can also lead to formation of MDA, HNE and isoprostanes. Hydroperoxides are reduced to alcohols by the enzyme glutathione peroxidase, which uses glutathione as a cofactor to do so. Glutathione is consequently oxidized, but the enzyme glutathione reductase reduces glutathione back to the reduced form. The action of reactive oxygen species (ROS) and of other RS is neutralized by several antioxidants, such as superoxide dismutase, catalase, glutathione, vitamins C and E, carotenoids and polyphenols. The action of non-enzymatic antioxidants (vitamins C and E, carotenoids and polyphenols) can be quantified using *in vitro* assays of antioxidant capacity. Finally, assays are also available to quantify the activity of enzymes used by the organism to repair damage to DNA, RNA or telomeres. Dashed lines indicate the fatty acid peroxidation chain. 8-OHG, 8-hydroxyguanosine; 8-OHdG, 8-hydroxy-2′-deoxyguanosine. Key: green, substrates that can be oxidized; grey, radical and non-radical reactive species; pink, antioxidant molecules (including damage repair enzymes); and red, oxidative damage compounds.

Finally, we highlight the fact that markers of oxidative status change in concert with other physiological end-points that are often used in conservation studies. Challenging conditions are, for example, known to affect glucocorticoid levels and the activity of immune cells, which are also functionally related to oxidative status. Hence, we make the point that merging information about stress hormones, immunocompetence and oxidative status would be important in order to provide better characterization of the response of natural animal populations to environmental perturbations and assess the relative importance of each physiological parameter in predicting the responses of populations to changes in environmental conditions. For example, if one physiological parameter stands out from the others in terms of predictive power, it could then be selected as the most relevant tool to be used in conservation of a given species. Principal component analysis can be a valuable tool to identify which parameters provide redundant information about the oxidative status and which ones are most sensitive to a certain environmental challenge (Costantini *et al.*, 2011b). For example, measurement of hydroperoxides, thiols and glutathione peroxidase might provide redundant information because these molecules interact with each other (Fig. 4). Hence, a better option can be to assess one or two of those markers and combine them with parameters that provide information on other components of oxidative status, such as damage to DNA or non-enzymatic antioxidant capacity. Likewise, it would be better to measure markers of damage to lipids, proteins and/or DNA rather than multiple markers of damage to one single class of macromolecules, because generation of damage to different macromolecules can occur independently from each other (Fig. 4). Biomarkers of oxidative damage and thiols (of protein and non-protein origin) are considered as the best proxy end-points of oxidative stress (Jones, 2006; Halliwell and Gutteridge, 2007; Sohal and Orr, 2012), while biomarkers of antioxidant defences (including damage repair enzymes) provide information about the way in which the organism is responding to an oxidative stressor. Interactions depicted in Fig. 4 can help in selection of the right number of biomarkers in order to obtain as much information as possible about the oxidative status of the organism.

## General conclusions

Conservation physiology is emerging as an essential component of conservation biology (Cooke *et al.*, 2013). It is increasingly recognized that physiological tools for the assessment of individual stress level or health are very important for decision-making in conservation programmes and for developing cause-and-effect relationships. In this review article, we have made the point that markers of oxidative status can provide additional and ecologically relevant information that can complement more conventional physiological parameters, such as stress hormones. Not only can markers of oxidative status provide a currency to quantify fitness costs, but they can also provide information about individual or population characteristics that other physiological parameters cannot detect. Acknowledging the significance of markers of oxidative status would therefore give conservation practitioners a new arrow in their quiver.
